# Effects of Deoxynivalenol Contamination on Growth Performance, Blood Biochemistry, Histology, Metabolomics, and the Microbiota: A Subacute Dose Oral Toxicity Study in Rats

**DOI:** 10.3390/ijms26073086

**Published:** 2025-03-27

**Authors:** Jinyoung Jeong, Junsik Kim, Minji Kim, Boram Lee, Cheolju Park, Minseok Kim

**Affiliations:** 1 Animal Nutrition and Physiology Division, National Institute of Animal Science, Wanju 55365, Republic of Korea; kkk940326@korea.kr (J.K.); mjkim00@korea.kr (M.K.); 2 Animal Biotechnology Division, National Institute of Animal Science, Wanju 55365, Republic of Korea; mir88@korea.kr; 3 Division of Animal Science, College of Agriculture and Life Sciences, Chonnam National University, Gwangju 61186, Republic of Korea; qkrcjfwn3@naver.com (C.P.); mkim2276@gmail.com (M.K.)

**Keywords:** deoxynivalenol, rat, microbiota, metabolism

## Abstract

Deoxynivalenol (DON), one of the most common mycotoxins, is frequently found in foods. This study investigated the effects of orally administered DON on the blood biochemical parameters, growth performance, histology, microbial composition, and metabolism of rats. After a 1-week adaptation period, 4-week-old rats were administered 0.9% saline (control), 1 mg/L DON (T1), 10 mg/L DON (T2), or 50 mg/L DON (T3) by gavage for 49 days. The DON-treated groups had significantly lower body weights than the control group (*p* < 0.05). Blood alkaline phosphatase, phosphate, cholesterol, amylase, and creatinine levels differed significantly between the DON-treated and control groups (*p* < 0.05). With increasing DON doses, fibrosis and apoptosis were observed in several tissues. In terms of metabolites, the bile acid biosynthesis pathway emerged as a potential biomarker, while the tryptophan metabolism pathway was found to be the most affected. The fecal microbiota showed significant differences in both alpha and beta diversity between the DON-treated and control groups (*p* < 0.05). In the cecal and fecal microbiota, the relative abundance of Firmicutes increased in the control and T1 groups, whereas Bacteroidota and Campylobacterota were more abundant in the T2 and T3 groups. In conclusion, our results showed that high DON exposure induces several dose-dependent adverse effects on rats.

## 1. Introduction

Mycotoxins are secondary compounds produced by fungi that can contaminate various crops, including maize, wheat, and barley, thus causing adverse effects on humans and animals upon consumption [1,2]. Deoxynivalenol (DON), a type B trichothecene produced by *Fusarium* species, is one of the most prevalent mycotoxins in cereal crops worldwide and frequently co-occurs in food and feed [2,3]. Increasing evidence suggests that that the contamination of food by DON can have several adverse effects on human and animal health, resulting in significant public health implications [4]. The European Food Safety Authority (EFSA) reported that DON was found in 75.2% of feed samples and 43.5% of food samples in the European Union [5]. Similarly, in the United States, contamination with DON was detected in 64.1% of the diets sampled and 65% of the multigrain diets [6,7]. In Asia, 98% of grain-based products and 96.4% of Chinese feed samples, as well as 95% of 494 South Korean feed samples, were found to be contaminated with DON [7,8,9].

Several studies in farm animals have shown that DON exerts complex toxic effects throughout the body [10]. DON-contaminated feed causes vomiting, diarrhea, and loss of appetite, leading to lower feed intake and efficiency in animals [11,12]. This can result in growth retardation and significant economic losses in livestock production. Additionally, when ingested, DON is rapidly absorbed and subsequently distributed throughout the body, targeting the blood, gastrointestinal tract, immune system, and lymphatic system [13]. Moreover, DON induces reactive oxygen species production, resulting in lipid peroxidation and the modulation of cellular antioxidant defense mechanisms [14]. These effects can cause histological changes such as fibrosis and apoptosis, as well as an imbalance in the intestinal microflora [15,16,17]. Finally, DON interferes with several metabolic processes, including glycolysis, protein biosynthesis, and cellular metabolism [18,19,20]. Furthermore, the side effects of DON vary depending on the dose [10]. Chronic low-dose exposure can lead to anorexia, immune dysfunction, and reproductive issues, whereas vomiting, leukocytosis, circulatory shock, reduced cardiac output, and ultimately death may result from acute high-dose exposure [21].

Generally, the susceptibility to DON toxicity in feeds is the highest in pigs [22], followed by mice, rats, poultry, and ruminants [22]. The rat model is particularly suitable for exploring the link between DON toxicity and biological systems as it facilitates a lifespan-wide analysis [23]. Thus, studies evaluating DON toxicity have primarily focused on rodents to establish the relationship between the dose and effect [15]. DON toxicity is dose-dependent, and assessing the various adverse effects at different doses and determining DON levels in feed samples are essential for assessing the risk to animals [13,24]. In this context, our previous study evaluated the effects of low-dose DON exposure (0.02 mg/L or 0.2 mg/L) on rats and identified negative effects throughout the body [25]. Building on this, in the present study, we aimed to evaluate the effects of high-dose DON on rats, comparing them with the effects of low-dose DON. Specifically, we investigated the effects of graded DON levels on growth performance, blood biochemistry, histological alterations, and the cecal microbiota in rats. We also assessed the relationship between metabolites, microorganisms, and growth performance (e.g., final body weight) in this study.

## 2. Results

### 2.1. Growth Performance

**Figure 1:**
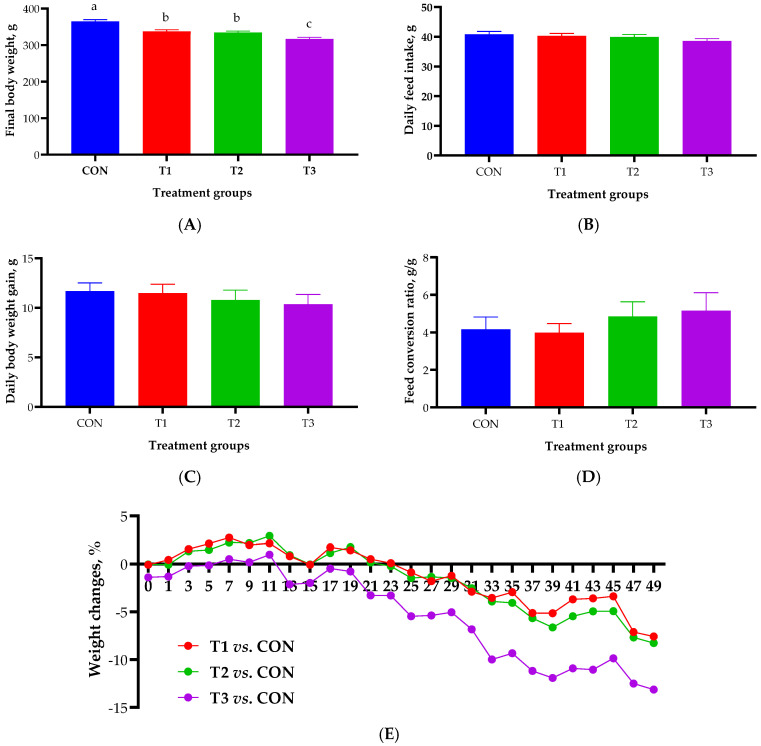
Growth performance of rats according to graded deoxynivalenol (DON) levels after 49 days of oral DON gavage in 4-week-old rats. (**A**) Final body weight. (**B**) Average daily feed intake. (**C**) Average daily body weight gain. (**D**) Feed conversion ratio. (**E**) Percentage of rat body weight changes. a, b, c Different superscript letters indicate significantly different values (*p* < 0.05). Treatment groups: CON, control (basal diet); T1, basal diet + 1 mg/L DON; T2, basal diet + 10 mg/L DON; T3, basal diet + 50 mg/L DON. Weight change (%) = (change in BW/control BW) × 100. BW, body weight.

The effect of oral DON gavage on growth performance over a 49-day period is shown in Figure 1. The final body weight was found to be significantly lower in the DON treatment groups than in the control group (365 ± 5.05 g). The T3 group exhibited the lowest final body weight among the DON-treated groups (317 ± 4.73 g), followed by the T1 (337 ± 4.94 g) and T2 groups (335 ± 3.83 g; Figure 1A). There were no significant differences in ADFI, ADG, or FCR among the control and the various DON-treated groups (Figure 1B–D). During the experimental period, the weight change in the DON treatment groups began to decline around day 25, with the most pronounced reduction observed in the T3 group (13.1%), followed by the T2 (8.26%) and T1 (7.58%) groups (Figure 1E).

### 2.2. Blood Biochemistry

**Figure 2:**
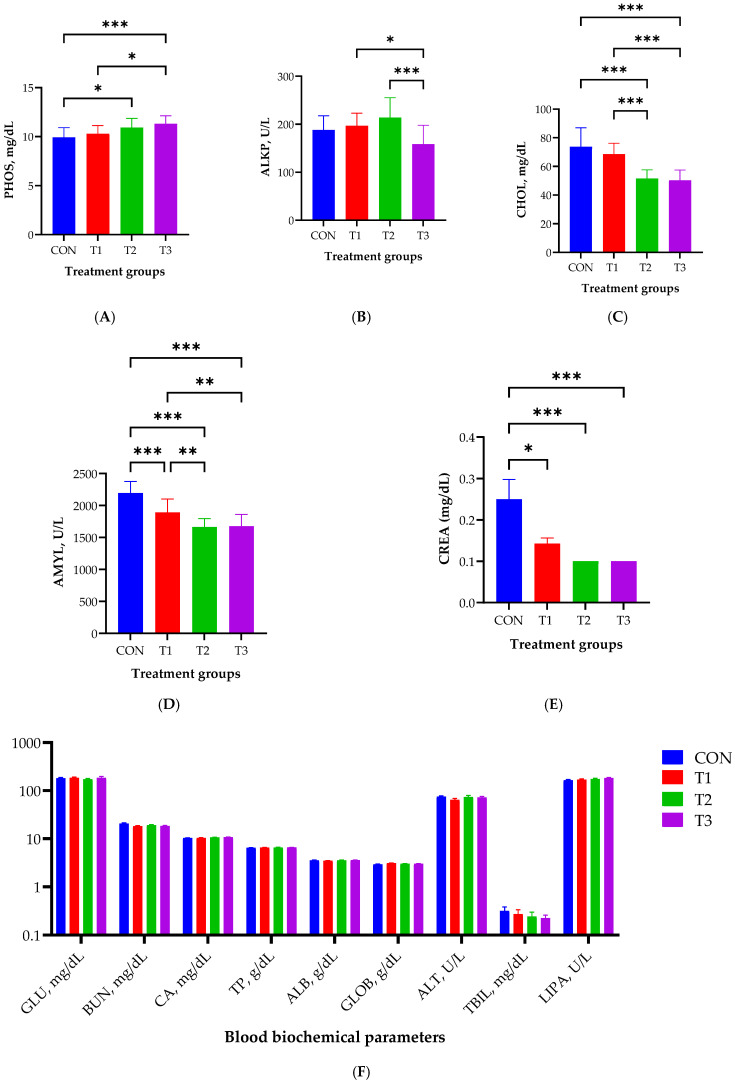
Effects of graded deoxynivalenol (DON) levels on blood biochemistry, including the levels of phosphate (PHOS, **A**), alkaline phosphatase (ALKP, **B**), cholesterol (CHOL, **C**), amylase (AMYL, **D**), creatinine (CREA, **E**), and nine biochemical parameters (**F**), in rats. Blood samples were collected from each rat via cardiac puncture using Vacutainer tubes without anticoagulants after 49 days of oral DON gavage. Treatment groups: CON, control (basal diet); T1, basal diet + 1 mg/L DON; T2, basal diet + 10 mg/L DON; T3, basal diet + 50 mg/L DON. *** <0.001, ** <0.01, and * <0.05.

Figure 2 illustrates the effect of oral DON gavage on blood biochemistry in rats over a 49-day period. The levels of PHOS were significantly elevated in the T2 (*p* < 0.05) and T3 (*p* < 0.001) groups compared with those in the control group, with the T3 group exhibiting the highest levels (Figure 2A). The lowest ALKP levels were observed in the T3 group (*p* < 0.001; Figure 2B). Cholesterol levels were significantly lower in the T2 and T3 groups than in the control and T1 groups (*p* < 0.001; Figure 2C). The levels of AMYL were significantly lower in the T1, T2, and T3 groups than in the control group (*p* < 0.001) and in the T2 and T3 groups than in the T1 group (*p* < 0.01, Figure 2D). CREA levels were significantly lower in the T1 (*p* < 0.05), T2 (*p* < 0.001), and T3 (*p* < 0.001) groups than in the control group (Figure 2E). Nine blood biochemical parameters did not exhibit statistically significant differences among the control and DON-treated groups (Figure 2F).

### 2.3. Histological Analysis in Different Tissues

**Figure 3:**
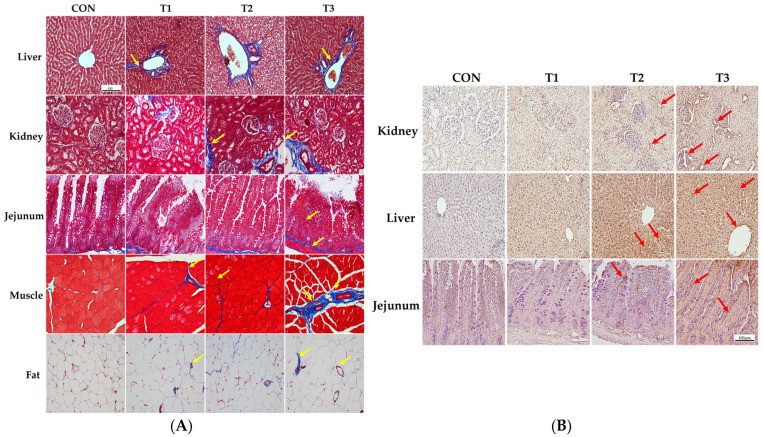
Histological analysis of the effects of graded levels of deoxynivalenol (DON) on rats. Histological images of the liver, kidney, jejunum, muscle, and adipose tissue were obtained by Masson’s trichrome staining to detect fibrosis after 49 days of oral DON administration (**A**), and apoptosis was detected by terminal deoxynucleotidyl transferase dUTP nick-end labeling (TUNEL) staining (**B**). As the DON concentration increased, fibrosis and TUNEL-positive staining gradually increased. Treatment groups: CON, control (basal diet); T1, basal diet + 1 mg/L DON; T2, basal diet + 10 mg/L DON; T3, basal diet + 50 mg/L DON. Arrows indicate staining for apoptosis (red) and fibrosis (yellow).

Figure 3 illustrates the effect of oral DON gavage on histological alterations in rats. The control group showed almost no signs of fibrosis in the liver tissue. However, in the T1 group, the blue staining indicated collagen accumulation in the portal vein and bile ducts. In the T2 group, collagen deposition extended to the surrounding tissues, and the degree of fibrosis was further aggravated in the T3 group. In kidney tissue, the control group displayed no signs of fibrosis and maintained normal histological structures. In contrast, the T1 group exhibited collagen deposition around the glomeruli, which became more prominent in the T2 group and extended to the surrounding tubular structures. In the T3 group, the degree of fibrosis was further aggravated, with widespread collagen deposition and a significant disruption of normal tissue architecture. In the jejunal tissue, DON treatment induced collagen deposition in the submucosal layer, with increasing DON concentrations leading to significant damage to the villus architecture. In the muscle tissue, minimal collagen deposition was observed between the muscle fibers in the T1 and T2 groups compared to the control group, suggesting early fibrosis. In the T3 group, collagen deposition became more pronounced, with a significant disruption of muscle fiber structures. Finally, in adipose tissue, DON treatment induced collagen accumulation between adipocytes, with accumulation increasing with increasing DON concentration, and extensive destruction of adipocyte morphology was observed in the T3 group (Figure 3A).

The results of the TUNEL staining performed to observe apoptosis in the kidney, liver, and jejunum are shown in Figure 3B. Our results indicated that clear signs of apoptosis were observed in the apical regions of the jejunal villi, as well as in renal cells and hepatocytes. The results showed that apoptosis indicated by TUNEL-positive staining increased with increasing DON concentrations in tissue cells compared to that in the control group in a dose-dependent manner.

### 2.4. Alpha and Beta Diversity of the Gut Microbiome

**Figure 4:**
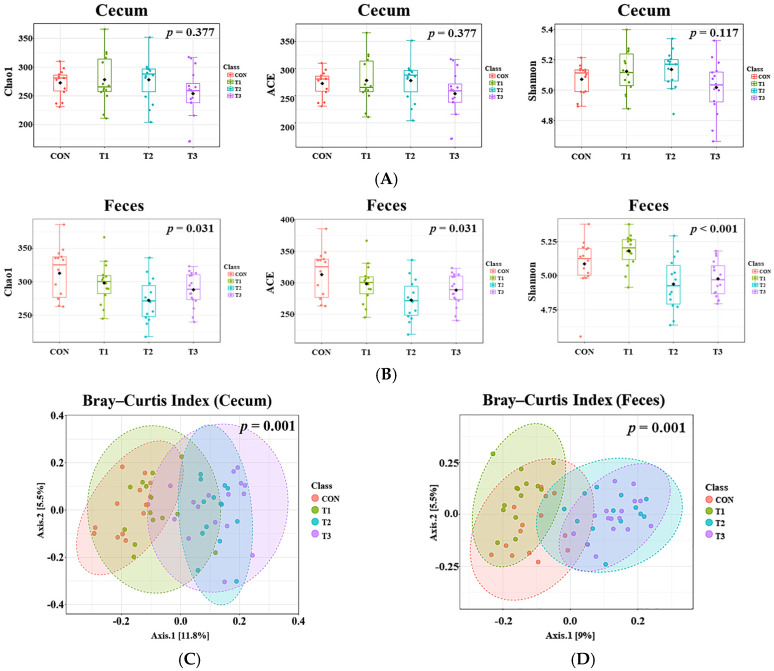
Analysis of alpha and beta diversity in rats according to graded deoxynivalenol (DON) levels. (**A**) Cecal alpha diversity measured by the Chao1, ACE and Shannon indices. (**B**) Fecal alpha diversity measured by the Chao 1 (Kruskal–Wallis, H = 8.8473, *p* = 0.03) ACE (Kruskal–Wallis, H = 8.8473, *p* = 0.03) and Shannon (Kruskal–Wallis, H = 16.927, *p <* 0.001) indices. The cecal microbiota showed no significant differences in any indices, whereas the fecal microbiota showed significant differences in all indices among the control and DON-treated groups (*p* < 0.05). Bray–Curtis index to assess differences in the microbial community composition in the rat cecum (**C**), R2 = 0.11304, *p* = 0.001) and feces (**D**), R2 = 0.13917, *p* = 0.001). Both the cecal and fecal microbiota showed significant differences among the control and DON-treated groups (*p* = 0.001). Treatment groups: CON, control (basal diet); T1, basal diet + 1 mg/L DON; T2, basal diet + 10 mg/L DON; T3, basal diet + 50 mg/L DON.

Alpha diversity, evaluated using the Chao 1, ACE, and Shannon indices, was measured to assess the diversity of the cecal and fecal microbiota in response to oral DON gavage (Figure 4A,B). The results revealed no significant differences in any diversity index for the cecal microbiota (Figure 4A), whereas the fecal microbiota exhibited significant differences across all indices among the control and DON-treated groups (*p* < 0.05). The richness of taxa within all DON-treated groups decreased, as indicated by the alpha diversity indices (Figure 4B). Furthermore, beta diversity was analyzed using the Bray–Curtis index to determine differences in the microbiome composition (Figure 4C). This analysis indicated significant differences in the Bray–Curtis index among the control and DON-treated groups for both the cecal and fecal microbiota (*p* = 0.001).

### 2.5. Cecal and Fecal Microbiota at the Phylum Level

**Figure 5:**
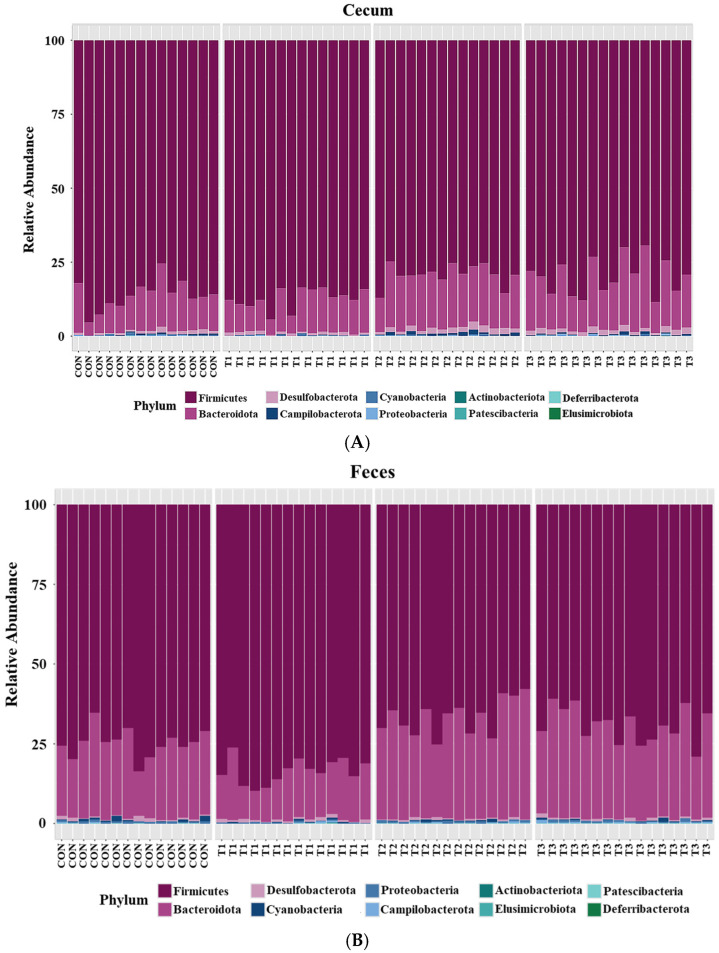
Microbial taxonomic bar plots at the phylum level in (**A**) the cecum and (**B**) feces of rats according to graded deoxynivalenol (DON) levels. Taxonomic compositions of the microbiota among control and DON-treated groups were compared based on the relative abundance (taxon reads/total reads in the cecum and feces). Treatment groups: CON, control (basal diet); T1, basal diet + 1 mg/L DON; T2, basal diet + 10 mg/L DON; T3, basal diet + 50 mg/L DON.

Taxonomic bar plots showing the mean relative abundances at the phylum level in the cecum and feces of the control and DON-treated groups are presented in Figure 5.

**Figure 6:**
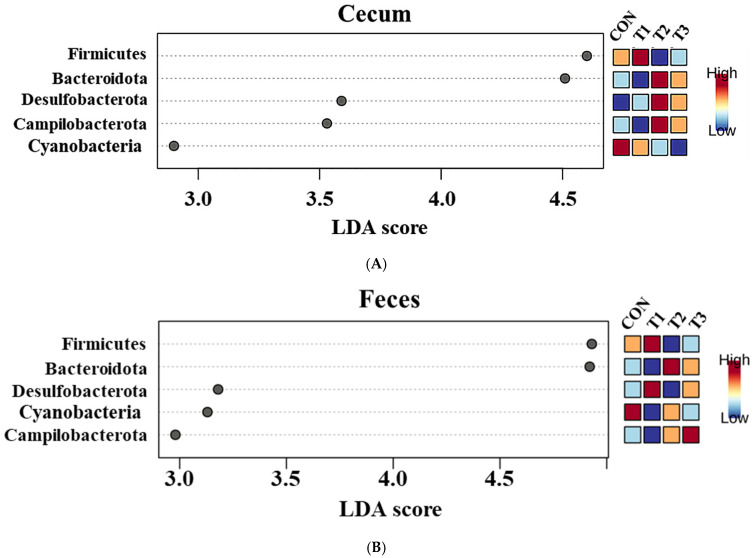
Graphical representation of the linear discriminant analysis (LDA) effect size (LEfSe) at the phylum level in the cecum (**A**) and feces (**B**) of rats according to graded deoxynivalenol (DON) levels. The horizontal bar represents the log10−transformed LDA score. Bacterial taxa were statistically significantly different (*p* < 0.05) in terms of relative abundance. Treatment groups: CON, control (basal diet); T1, basal diet + 1 mg/L DON; T2, basal diet + 10 mg/L DON; T3, basal diet + 50 mg/L DON.

Firmicutes (cecum: 83.3%, feces: 73.6%) was the most abundant phylum in both the cecum and feces across among the control and DON-treated groups, followed by Bacteroidota (cecum: 14.8%, feces: 24.9%). At the phylum level, a LEfSe was conducted to identify the differentially abundant taxa among the control and DON-treated groups (Figure 6).

In the cecum, LEfSe revealed that the T2 and T3 groups exhibited higher relative abundances of Bacteroidota (control: 12.20%, T1: 11.26%, T2: 17.76%, T3: 17.51%), Desulfobacterota (control: 0.78%, T1: 0.79%, T2: 1.56%, T3: 1.49%), and Campylobacterota (control: 0.28%, T1: 0.10%, T2: 0.77%, T3: 0.43%). Conversely, the control and T1 groups were characterized by higher relative abundances of Firmicutes (control: 86.28%, T1: 87.49%, T2: 79.58%, and T3: 80.24%) and Cyanobacteria (control: 0.28%, T1: 0.24%, T2: 0.21%, and T3: 0.12%). In the feces, LEfSe indicated that the T2 and T3 groups had higher relative abundances of Bacteroidota (control: 23.40%, T1: 14.90%, T2: 31.60%, T3: 29.06%) and Campylobacterota (control: 0.15%, T1: 0.14%, T2: 0.24%, T3: 0.33%). The control and T1 groups were characterized by a higher relative abundance of Firmicutes (control: 74.97%; T1: 83.84%, T2: 67.01%, T3: 69.34%).

### 2.6. Cecal and Fecal Microbiota at the Genus Level

**Figure 7:**
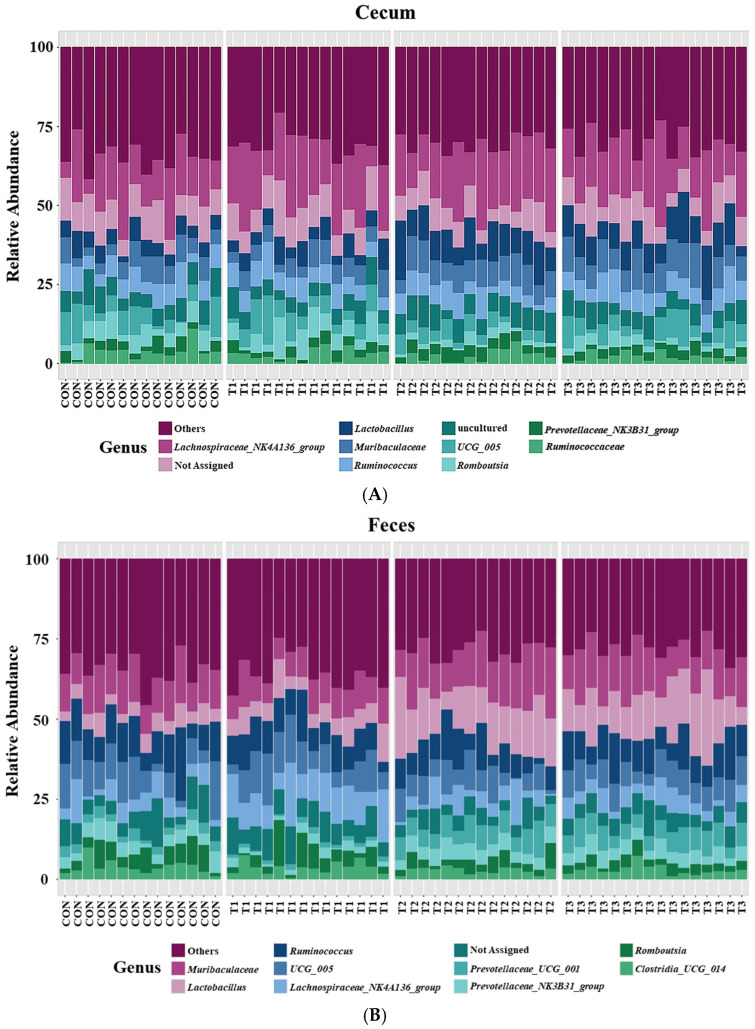
Microbial taxonomic bar plots at the genus level in the cecum (**A**) and feces (**B**) of rats according to graded deoxynivalenol (DON) levels. Taxonomic compositions of the microbiota among the control and DON-treated groups were compared based on the relative abundances (taxon reads/total reads in the cecum and feces). Treatment groups: CON, control (basal diet); T1, basal diet + 1 mg/L DON; T2, basal diet + 10 mg/L DON; T3, basal diet + 50 mg/L DON.

Taxonomic bar plots showing the mean relative abundances at the genus level in the cecum and feces of the control and DON-treated groups are presented in Figure 7.

In cecal samples, the *Lachnospiraceae NK4A136* group (17.8%) was the most abundant genus, followed by *Lactobacillus* (7.8%), *Muribaculaceae* (7.1%), *Ruminococcus* (6.3%), *UCG 005* (5.2%), *Romboutsia* (3.6%), *Prevotellaceae NKB31* group (3.0%), and *Ruminococcaceae* (2.6%). In fecal samples, *Muribaculaceae* (12.4%) was the most abundant genus, followed by *Lactobacillus* (10.2%), *Ruminococcus* (9.5%), *UCG 005* (8.0%), *Lachnospiraceae NK4A136* group (6.8%), *Prevotellaceae UCG 001* (4.2%), *Prevotellaceae NKB31* group (3.9%), *Romboutsia* (3.7%), and *Clostridia UCG 014* (3.5%).

**Figure 8:**
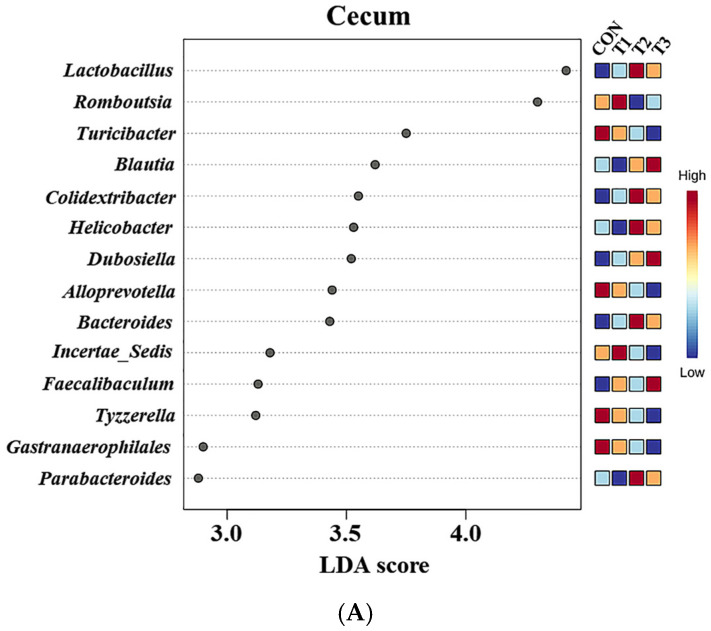

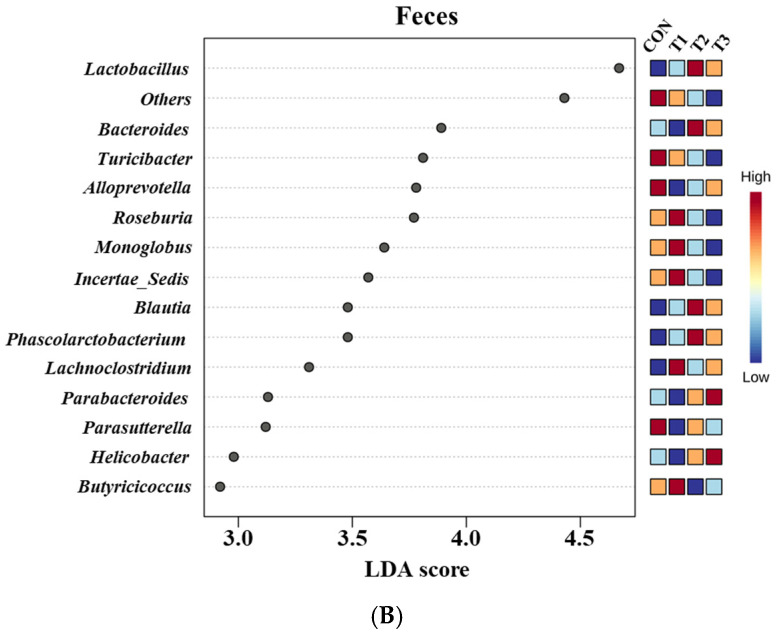
Graphical representations of the linear discriminant analysis (LDA) effect size (LEfSe) at the genus level in the cecum (**A**) and feces (**B**) of rats according to graded deoxynivalenol (DON) levels. The horizontal bar represents the log10-transformed LDA score. The bacterial taxa were statistically significantly different (*p* < 0.05) in terms of relative abundance. Treatment groups: CON, control (basal diet); T1, basal diet + 1 mg/L DON; T2, basal diet + 10 mg/L DON; T3, basal diet + 50 mg/L DON.

At the genus level, LEfSe was performed to identify the differentially abundant taxa among the control and DON-treated groups (Figure 8). In cecal samples, LEfSe revealed that the T2 and T3 groups exhibited increased relative abundances of *Helicobacter* (control: 0.28%, T1: 0.10%, T2: 0.77%, and T3: 0.43%), *Lactobacillus* (control: 5.14%, T1: 5.63%, T2: 10.34%, and T3: 9.73%), *Bacteroides* (control: 0.23%, T1: 0.24%, T2: 0.77%, and T3: 0.59%), *Colidextribacter* (control: 0.86%, T1: 1.05%, T2: 1.56%, and T3: 1.40%), *Parabacteroides* (control: 0.12%, T1: 0.12%, T2: 0.27%, and T3: 0.27%), and *Blautia* (control: 0.36%, T1: 0.33%, T2: 0.76%, and T3: 1.16%). Conversely, the control and T1 groups were characterized by increased relative abundances of *Romboutsia* (control: 4.68%, T1: 5.72%, T2: 1.75%, T3: 2.33%), *Tyzzerella* (control: 0.31%, T1: 0.32%, T2: 0.08%, and T3: 0.06%), *Alloprevotella* (control: 0.94%, T1: 0.58%, T2: 0.39%, and T3: 0.39%), and *Gastranaerophilales* (control: 0.28%, T1: 0.24%, T2: 0.21%, and T3: 0.12%). In fecal samples, LEfSe indicated that the T2 and T3 groups had elevated relative abundances of *Lactobacillus* (control: 5.03%, T1: 6.59%, T2: 14.43%, and T3: 14.06%), *Parabacteroides* (control: 0.25%, T1: 0.23%, T2: 0.48%, and T3: 0.50%), *Bacteroides* (control: 0.78%, T1: 0.63%, T2: 2.18%, and T3: 1.63%), *Helicobacter* (control: 0.15%, T1: 0.14%, T2: 0.24%, and T3: 0.33%), and *Blautia* (control: 0.29%, T1: 0.31%, T2: 0.88%, and T3: 0.86%). The control and T1 groups were characterized by increased relative abundances of *Roseburia* and *Turicibacter*. Additionally, the relative abundance of *Alloprevotella* (control: 1.71%, T1: 0.50%, T2: 0.63%, and T3: 0.70%) was higher in the control group than in the DON-treated groups.

### 2.7. Metabolomic Profiling

**Figure 9:**
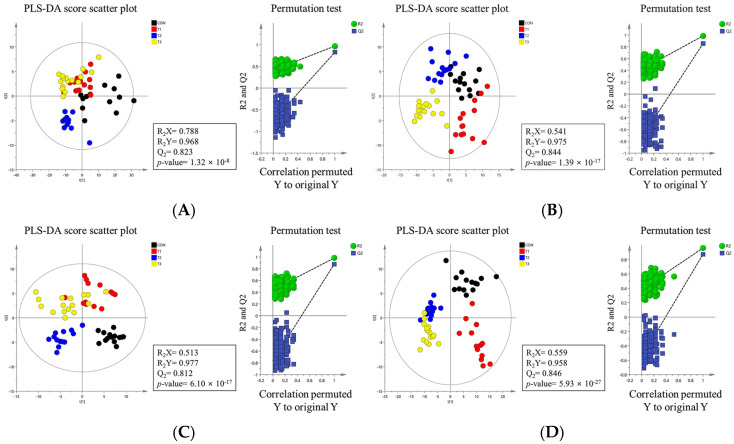

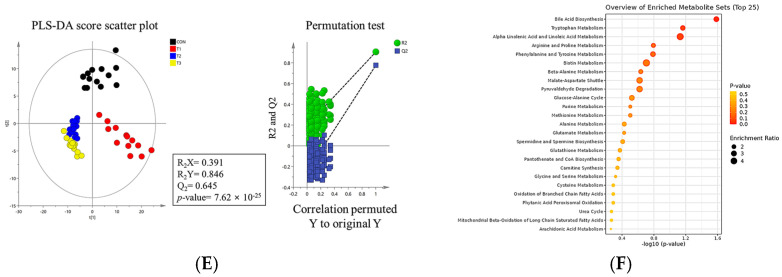
Metabolite profiling of rats according to graded levels of deoxynivalenol (DON). Partial least discriminant analysis (PLS-DA) scatter plots and permutation plots of blood (**A**), liver (**B**), kidney (**C**), cecum (**D**), and feces (**E**). Metabolic pathways (**F**). A 95% confidence interval was used to define deviations in the score plots. Clear clustering (*p* < 0.05) was observed for metabolites in DON−treated rats compared to the control group. Treatment groups: CON, control (basal diet); T1, basal diet + 1 mg/L DON; T2, basal diet + 10 mg/L DON; T3, basal diet + 50 mg/L DON.

To understand the metabolic impact of DON toxicity at different levels, we characterized the metabolites in the blood, liver, kidney, cecum, and feces of rats using liquid chromatography–mass spectrometry. Partial Least Squares Discriminant Analysis (PLS-DA) indicated that metabolites in the DON-treated and control groups were significantly separated in the blood, liver, kidney, cecum, and feces (Figure 9).

We conducted additional statistical analyses on DON-contaminated rats to identify potential tissue-specific biomarkers. Metabolites in tissues, including blood, liver, kidney, cecum, and feces, showed significant changes, as indicated by VIP scores >1.0 and *p* < 0.05. In blood, phenylalanine, tryptophan, benzoarachidonic acid/tetracosahexaenoic acid, LPC (14:0), LPC (22:6), LPC (20:4), LPC (20:5), LPC (18:2), LPC (22:5), LPC (20:3), LPC (22:4), LPC (18:1), LPC (20:2), LPC (15:0), LPC (18:0), LPC (16:0), and LPC (17:0) were significantly changed among the control and DON-treated groups. In the liver, xanthine, 2′,3′-anhydroadenosine/deoxy-cycloadenosine, taurocholic acid, glycocholic acid, LPC (14:0), LPC (16:1), LPC (20:4), palmitoylcarnitine, LPE (16:0), LPC (16:0), stearoylcarnitine, LPC (18:1), LPE (18:1), LPE (18:0), LPC (18:0), 11-eicosenamide, and isobutyl-2,4-octadecadienamide were significantly changed among the control and DON-treated groups. In the kidneys, neuraminic acid, succinyladenosine, ethyl docosahexenoate, lauroylcarnitine, 9-octadecenamide, myristoyl-L-carnitine, LPC (14:0), hexadecenoylcarnitine, palmitoylcarnitine, oleoylcarnitine, eicosadienoylcarnitine, LPC (10:3), stearoylcarnitine, LPC (P-16:0), and icosanoylcarnitine levels were significantly different among the groups. In cecum, phenylglyoxylic acid/piperonal, phenylalanine, carboxyindole, 5-methylthioadenosine, 3-hydroxy-3-methyloxindole, stercobilin, hydroxycholic acid, cholic acid/ursocholic acid/muricholic acid, cholic acid/ursocholic acid/muricholic acid, chenodeocycholic acid/deoxycholic acid/hyodeoxycholic acid, ketodeocycholic acid/nutriacholic acid/alpha-apcholic acid, chenodeoxycholic acid/deoxycholic acid/hyodeoxycholic acid, and ketodeoxycholic acid/nutriacholic acid/alpha-apocholic acid were significantly changed among treatment groups.

**Figure 10:**
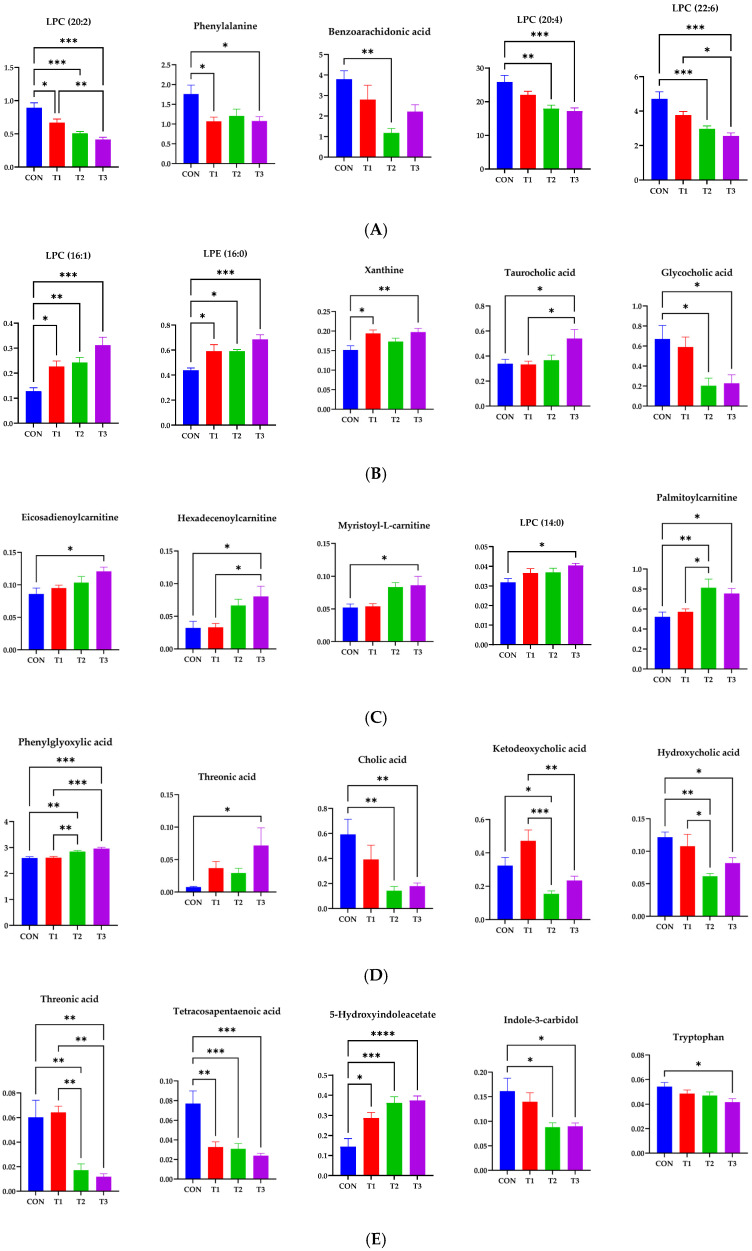
Representative bar graphs of the variable importance in projection values of metabolites in blood and different tissues. (**A**) Blood, (**B**) liver, (**C**) kidney, (**D**) cecum, and (**E**) feces, respectively. Metabolite levels in different samples were significantly different using ANOVA based on Tukey’s test for comparing means. Treatment groups: CON, control (basal diet); T1, basal diet + 1 mg/L DON; T2, basal diet + 10 mg/L DON; T3, basal diet + 50 mg/L DON. **** <0.0001, *** <0.001, ** <0.01, and * <0.05.

In feces, phenylglyoxylic acid/piperonal, thereonic acid, 3-hydroxy-3-methyloxindole, kynurenic acid, 5-hydroxyindoleacetate, indole-3-carbidol, stercobilin, alpha-aspartyl-L-phenylalanine, cholic acid/ursocholic acid/muricholic acid, cholic acid/ursocholic acid/muricholic acid, chenodeoxycholic acid/deoxycholic acid/hyodeoxycholic acid, ketodeoxycholic acid/nutriacholic acid/alpha-apocholic acid, and chenodeocycholic acid/deoxycholic acid/hyodeoxycholic acid were significantly changed among the treatment groups. Some of the most significant candidate metabolites are shown (Figure 10). In five different samples, including blood, there were 34 increases and 48 decreases in the DON group.

### 2.8. Simple Linear Regression Analysis

**Figure 11:**
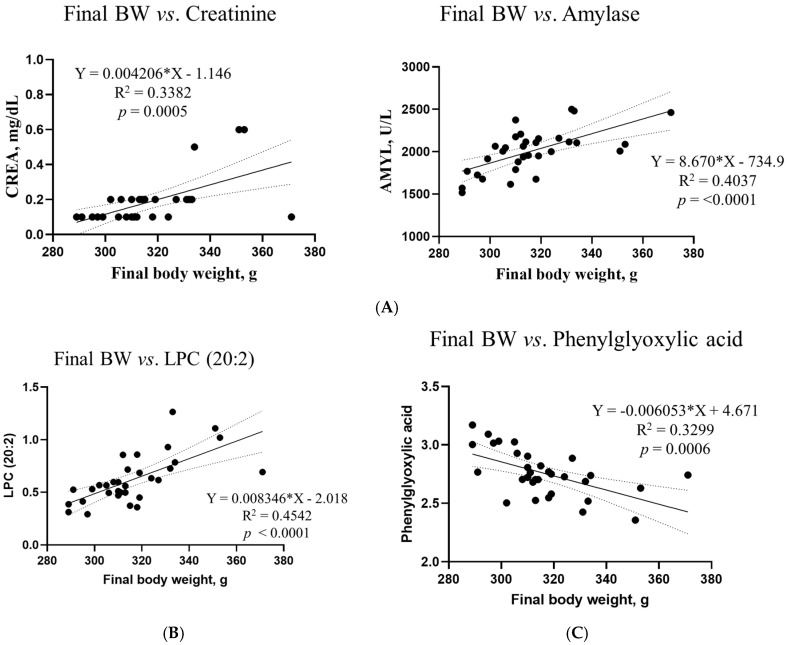

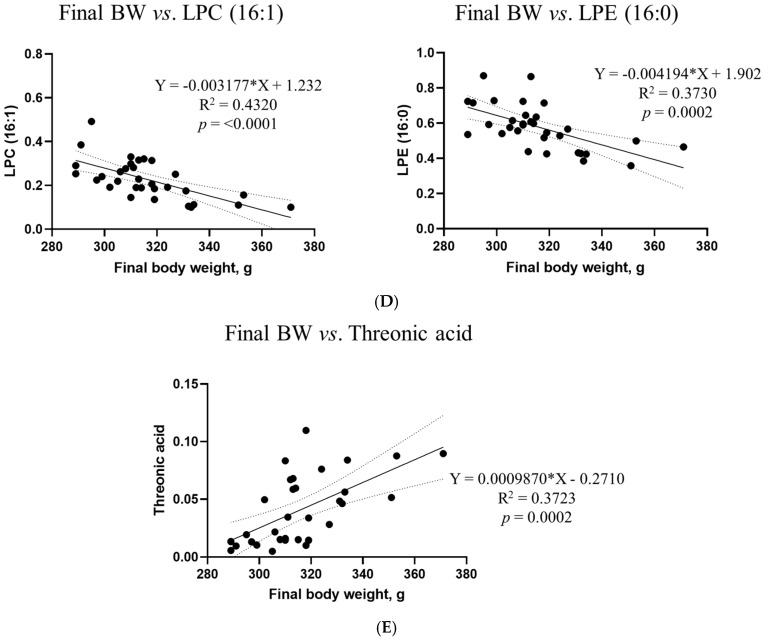
Simple linear regression analysis of the associations between the final body weight and biochemical parameters and metabolites in the DON treatment groups. (**A**) Blood creatinine and amylase levels. For metabolites, (**B**) LPC (20:2) in blood, (**C**) phenylglyoxylic acid in the cecum, (**D**) LPC (16:1) and LPE (16:0) in the liver, and (**E**) threonic acid in feces showed correlations. GraphPad Prism software (version 9.5.1) was used to calculate the correlation coefficients and *p*−values. The conditions for the linear regression analysis are as follows: VIP > 1, *p* < 0.05, and R2 > 0.3.

Based on VIP >1, *p* < 0.05, and R2 >0.3, simple linear regression analyses were performed between the final body weight and blood biochemical parameters, metabolites, and microbiota (Figure 11). However, no differences in the microbiota composition or renal metabolites were found in this study. Correlations between the final body weight, blood biochemical parameters, and metabolites were consistent; creatinine (R2 = 0.3382, *p* = 0.0005) and amylase (R2 = 0.4037, *p* < 0.0001) were among the biochemical parameters. Among the metabolites, LPC (20:2) (R2 = 0.4542, *p* < 0.0001) in blood, LPC (16:1) (R2 = 0.4320, *p* < 0.0001) and LPE (16:0) ‘(R2 = 0.3730, *p* = 0.0002) in the liver, phenylglyoxylic acid (R2 = 0.3299, *p* = 0.0006) in the cecum, and threonic acid (R2 = 0.3723, *p* = 0.0002) in feces exhibited correlations.

## 3. Discussion

In this study, we investigated the effects of low-to-high doses of DON on growth performance, blood biochemistry, histological changes, metabolomic profiles, and the cecal microbiota composition in 4-week-old rats over a seven-week period. The effects of DON on the body can vary significantly depending on the dosage administered [22]. Therefore, we investigated the toxicity of each dose and performed comparative analyses. In the present study, DON was orally administered to rats at doses of 1, 10, and 50 mg/L. To the best of our knowledge, few studies have investigated the adverse effects of oral administration of high doses of DON (>10 mg/L) on the body of rats. The T1 treatment dose of 1 mg/L of DON was based on the maximum contamination limit recommended by the FDA for finished wheat products intended for human consumption [26]. Similarly, the T2 treatment dose of 10 mg/L DON was aligned with the FDA’s recommended maximum contamination limit for feed grains and grain by-products [26]. As DON contamination in feed can range from 0 to 50 mg/kg [27], the T3 treatment dose was set at 50 mg/L to assess the toxicity of high DON doses.

Several studies have reported that DON negatively affects animal growth performance [14,28,29]. Growth retardation is an important adverse effect of DON treatment. In livestock, it can lead to significant economic losses due to associated symptoms, such as diarrhea, vomiting, and reduced feed intake [24,30]. In this study, all DON treatments resulted in a significant reduction in the final body weight of rats. Compared with the control group, body weight decreased by 7.7% in the T1 group, 8.2% in the T2 group, and 13.2% in the T3 group, indicating a dose-dependent effect. Low-dose DON toxicity led to growth retardation, whereas higher doses exacerbated this effect. Rats exhibit intermediate susceptibility to DON toxicity compared to other animals. These differences in susceptibility may be attributed to differences in metabolism, absorption, distribution, and elimination of DON across species [22]. This suggests that animals that are more sensitive to DON toxicity than rats, such as pigs, may experience growth retardation even at low doses. However, no effects of DON toxicity on daily feed intake or the feed conversion ratio were observed in our results. In general, approximately 85% of weight loss due to mycotoxicosis is due to reduced feed intake [31]. However, DON not only reduces feed intake but also damages the intestinal wall, impairs nutrient absorption and utilization, and may interfere with organ function, leading to inefficient nutrient utilization [28,32]. Therefore, to determine the exact cause of weight loss, further studies that consider multiple factors related to DON are needed.

In the present study, serum levels of ALKP, AMYL, CHOL, CREA, and PHOS were affected by DON gavage in rats. Serum ALKP is secreted by mucosal cells lining the biliary tract of the liver and can leak into the bloodstream when liver cells are damaged [33,34]. DON can cause severe liver damage because the liver is the primary organ responsible for detoxifying and metabolizing mycotoxins [35]. Consequently, the decrease in serum ALKP levels observed in the T3 group may indicate liver damage resulting from DON-induced systemic toxicity, possibly due to the abnormal excretion of hepatic metabolites [36]. CHOL, a key component of lipid metabolism, is mainly synthesized de novo in the liver [37]. Maintenance of CHOL homeostasis is critical for mitigating DON-induced liver injury. However, mycotoxins can inhibit CHOL production by modulating the genes associated with lipid metabolism [37,38], which may explain the reduced CHOL levels observed in the high-dose DON group. AMYL is a digestive enzyme that catalyzes the hydrolysis of glycogen to produce maltose and glucose, which provide energy for the body [39]. The consumption of DON damages the intestinal mucosa and increases intestinal permeability, leading to reduced nutrient absorption and impaired digestive organ function [40]. This could lead to the DON-induced inhibition of digestive enzyme secretion. CREA is used to assess glomerular filtration and is an important indicator of the severity of kidney damage [41]. High blood phosphate levels are associated with chronic kidney disease [42]. DON can induce oxidative stress in the body, leading to oxidative damage to the kidneys, which may explain our findings [41].

Based on the findings of this study, histological alterations, including apoptosis and fibrosis, were observed in various organs, including the kidneys, liver, jejunum, muscle, and adipose tissue, all of which exhibited dose-dependent effects. DON is absorbed in large amounts upon ingestion and is rapidly distributed throughout the tissues, leading to adverse effects on multiple organs [13]. Although the mechanism by which DON causes renal damage is not fully understood, it is excreted through the kidneys into the urine, where it may induce oxidative stress and mitochondrial damage [43]. As mentioned earlier, the liver is a major target of DON toxicity because it plays a key role in detoxifying and metabolizing mycotoxins after ingestion [35]. Additionally, 51% of ingested DON is absorbed in the small intestine, with the jejunum appearing particularly vulnerable to DON toxicity [44,45], making it severely affected. The histological alterations observed in this study may be closely linked to the oxidative stress induced by DON. DON causes oxidative stress by promoting the accumulation of reactive oxygen species (ROS) and impairing the function of multiple antioxidant enzymes, including superoxide dismutase (SOD), catalase (CAT), and malondialdehyde (MDA) [43,46]. Oxidative stress can damage mitochondrial membranes and structures in the liver tissue, leading to DON-induced apoptosis [34]. Furthermore, DON-induced oxidative stress increases the expression of apoptosis-related genes and proteins, such as interleukin-1 beta (IL-1β), cyclooxgenase-2 (COX-2), interleukin-6 (IL-6), tumor necrosis factor-alpha (TNF-α), caspase-3, caspase-8, caspase-9, caspase-12, and BAX in intestinal epithelial cells and renal tissue [43,45]. Furthermore, oxidative stress can lead to fibrosis, which is characterized by the excessive accumulation of matrix and connective tissue components [47,48]. Oxidative stress upregulates fibrotic genes such as tumor growth factor beta1 (TGF-β1), driving fibrosis by promoting the accumulation of extracellular matrix components [49]. Additionally, cell damage caused by oxidative stress can initiate an inflammatory response mediated by cytokines, and sustained activation of this response may result in tissue fibrosis [49,50,51]. The histological alterations observed in our study resulting from DON exposure may contribute to the development of chronic diseases and provide evidence supporting the weight loss described earlier. These changes have the potential to cause organ dysfunction and mortality in severe cases.

The intestinal microbiota is crucial for animal health, as it performs nutritional and protective functions by preventing pathogen fecalization and supporting normal mucosal immunity [19]. However, the intestine is the primary target of DON exposure, which can impair absorptive functions, disrupt barrier integrity and immune responses, induce microbiome imbalances, and compromise overall gut health [40,52]. In this study, the alpha diversity indices showed no differences between the cecal microbiota of the control and DON-treated groups, whereas those among the fecal microbiota showed significant differences. The beta diversity analysis using the Bray–Curtis index revealed distinct clustering among the control and DON-treated groups in both the cecal and fecal microbiota. Consequently, our findings suggest that DON exposure significantly alters the microbial community composition. However, the different patterns observed between the cecal and fecal microbiota may be due to regional differences in the gut microbiota composition that affect functional diversity. The gastrointestinal tract exhibits significant variations in nutritional and chemical compositions, water and oxygen contents, temperature, and pH levels, depending on its specific location [53]. In this regard, our study focused on the diversity of the various segments of the gastrointestinal tract.

Firmicutes and Bacteroidota were the dominant phyla in both the cecum and feces, regardless of DON treatment, which is consistent with findings from previous rat studies [54,55]. In the cecal and fecal microbiota of rats, the relative abundance of Firmicutes was increased in the control and low-dose DON groups (1 mg/L). Firmicutes contributes to host energy metabolism by breaking down complex carbohydrates into short-chain fatty acids (SCFAs), which are associated with weight gain [56,57]. The abundance of cyanobacteria increased in the cecal microbiota of the low-dose DON group, and Drobac et al. [58] reported that certain species of cyanobacteria produce toxic metabolites known as cyanotoxins. However, Zhang et al. [59] highlighted that cyanotoxin toxicity varies by strain, suggesting that positive identification alone may not reliably predict risk levels. In contrast, the high-dose DON groups (≥10 mg/L) showed increased relative abundances of Bacteroidota and Campylobacterota in both the cecal and fecal microbiota. Additionally, the relative abundance of Desulfobacterota increased in the cecal microbiota of the high-dose DON group. Although Bacteroidota is a major component of the gut microbiota in mice, an increase in this phylum has been linked to weight loss [60]. Campylobacterota and Desulfobacterota, which are pathogenic in rats, contribute to adverse health effects [61]. An increased Campylobacter abundance can compromise the intestinal barrier function, causing diarrhea, and has been associated with liver disease [61,62]. Elevated Desulfobacterota levels have been associated with stress and depression, and their inhibition improves motor impairments and neuronal deficits [63].

At the genus level, the relative abundances of *Lactobacillus*, *Helicobacter*, *Bacteroides*, *Parabacteroides*, and *Blautia* in the cecal and fecal microbiota were elevated in the high-dose DON group. Although some species within *Lactobacillus* can cause pathogenic conditions, including bacteremia, hepatic abscesses, and sepsis [64,65], the genus as a whole is widely recognized as beneficial. Therefore, further research is needed to clarify the effects on DON on this genus. Similarly, certain species within the genus *Helicobacter*, such as *Helicobacter bilis* and *Helicobacter pylori*, cause pathogenic diseases. These diseases include pancreatitis, gastritis, hepatitis, cholangitis, and immune system abnormalities in rats [66,67]. *Bacteroides* produces pathogenic species that can cause appendicitis and inflammatory bowel disease [68]. Additionally, certain species are associated with weight loss [69]. An increase in *Parabacteroides* may affect the levels of reproductive hormones in rats, potentially causing reproductive toxicity, and may also be associated with suppressed weight gain [70,71]. The abundance of *Blautia*, a genus of anaerobic opportunistic pathogens, is associated with gastrotoxicity in rats, potentially contributing to diarrhea and inflammatory bowel disease [72,73].

Furthermore, in the cecal microbiota of the high-dose DON group, *Colidextribacter* exhibited an increased relative abundance, whereas *Romboutsia*, *Tyzzerella*, and *Gastranaerophilales* demonstrated decreased relative abundances. Decreases in the relative abundances of *Roseburia* and *Turicibacter* were also observed in the fecal microbiota of the high-dose DON group. An increased abundance of *Tyzzerella* is associated with increased body weight in rats [74]. *Colidextribacter* induces oxidative stress and impairs barrier function [75]. *Romboutsia* is abundant in the healthy intestinal mucosa and promotes the production of SCFAs [76,77]. Additionally, an increase in the relative abundance of *Romboutsia* may be associated with weight gain in rats [76]. *Gastranaerophilales* is a potential intestinal probiotic that supports the digestion and absorption of diverse sugars, produces butyrate, and exerts anti-inflammatory and immunomodulatory functions [78]. *Roseburia* is a potential marker of gut health. It is also negatively correlated with various gastrointestinal diseases, including inflammatory bowel disease, irritable bowel syndrome, and fecal cancer [79]. *Roseburia* also produces butyric acid, which exhibits immunosuppressive and anti-inflammatory properties [80]. A decrease in the relative abundance of *Turicibacter* is associated with renal damage in rats and is involved in host bile acid and lipid metabolism [81]. Notably, the relative abundance of *Alloprevotella* was lower in all DON-treated groups than in the control group. *Alloprevotella* produces beneficial short-chain fatty acids (SCFAs) that provide energy to intestinal cells, protect the intestinal wall, and enhance digestion in animals [82,83]. Therefore, the changes in the relative abundances of microorganisms among the control and DON-treated groups observed in this study may offer insights into body weight variations and multi-organ damage.

In the present study, the metabolites investigated varied among the sampled tissues, highlighting the usefulness of mycotoxin metabolomics in clarifying the link between the diet and contamination. Notably, we used a comprehensive design that accounted for sex, age, and breed while monitoring growth performance and biochemical, histological, and microbiome parameters in various tissues. Phenylalanine and tryptophan are essential for amino acid biosynthesis and are essential dietary components of proteins and enzymes. Phenylalanine is metabolized to tyrosine, which is converted into other compounds involved in various biological processes. Tryptophan has several important roles in the human body. Carbohydrates increase tryptophan availability in the brain, while protein intake decreases it. This suggests that dietary intake alters tryptophan availability. A transcriptomic analysis has identified enriched KEGG pathways for phenylalanine, tyrosine, and tryptophan biosynthesis in intestinal tissue changes caused by probiotic strains [84]. Fungal toxins significantly affect xanthine metabolism. These toxins can inhibit enzymes, such as xanthine oxidase, which is crucial for converting xanthine into uric acid. This inhibition can lead to the accumulation of xanthine and hypoxanthine, potentially causing disorders related to purine metabolism. Mycotoxins disrupt the normal metabolism of xanthine by inhibiting enzymes, such as xanthine oxidase. This can lead to the accumulation of uric acid, disrupting purine metabolism, and resulting in health problems. Some of them can damage tissues and organs, exacerbating the effects of impaired metabolism [85]. In the present study, we also reported that ether lipid metabolism, amino acid metabolism, and purine metabolism affected the pathways targeted as potential biomarkers of DON in rats. Among these pathways, bile acid biosynthesis was the most prominent pathway identified in this study. To improve the accuracy and reliability of our results, future research should involve comprehensive data analysis and problem-solving approaches, including functional characterization and pathway analysis.

## 4. Materials and Methods

### 4.1. Ethics Statements

The Institutional Animal Care and Use Committee of the National Institute of Animal Science, Korea (No. NIAS-2022-0546), reviewed and approved all the experimental procedures.

### 4.2. Animal and Study Design

Three-week-old male Sprague–Dawley (SD) rats used in this study were obtained from Koatech (Pyeongtaek, Republic of Korea). Fifty-eight SD rats were housed in individual cages (27.7 cm in width × 42.3 cm in length × 19.4 cm high). After one week of acclimatization, mice were maintained at a room temperature of 23 ± 2 °C, relative humidity of 55 ± 5%, and a 12-h light/dark cycle during the experiment. The animals were divided into four groups: (1) a control group (n = 14) fed a basal diet + 0.9% saline; (2) T1 group (n = 14) fed a basal diet + 1 mg/L DON (actual absorption amount, 0.0995 mg/kg feed/100 g BW); (3) T2 group (n = 14) fed a basal diet + 10 mg/L DON (actual absorption amount, 0.9954 mg/kg feed/100 g BW); and (4) T3 group (n = 16) fed a basal diet + 50 mg/L DON (actual absorption amount, 4.9771 mg/kg feed/100 g BW). The dosage capacity also increased with increasing body weight. In this study, we designed experimental groups to measure the in vivo changes in the concentration to determine their correlation with the in vivo changes in rats, based on a previous study. Animals were orally administered 0.9% saline or DON mixed with 0.9% saline daily for 49 days. Rats had ad libitum access to food and water throughout the study. Powdered DON (TripleBond, Guelph, ON, Canada) was thoroughly mixed with an organic solvent (95% ethyl alcohol; Lab Alley, Austin, TX, USA). The animals were anesthetized using CO_2_. Tissues such as the liver, kidney, jejunum, muscle, abdominal fat, and blood from the heart were quickly collected. The collected tissues, cecal contents, and feces were also immediately frozen in liquid nitrogen and stored at −80 °C (UniFreez U500, Daehan Scientific, Wonju, Republic of Korea). For the histological analysis, tissues were fixed with 10% neutral buffered formalin (NBF; Sigma-Aldrich, St. Louis, MO, USA). Growth characteristics, including average daily gain (ADG), average daily feed intake (ADFI), and feed conversion ratio (FCR), were calculated based on a previous study [25].

### 4.3. Blood Biochemical Analysis

On day 49, blood was collected from each rat via cardiac puncture into anticoagulant-free tubes. Serum was centrifuged at 700× *g* for 15 min at 4 °C and then stored at −80 °C. All blood parameters, including glucose, creatinine (CREA), blood urea nitrogen, phosphate (PHOS), calcium, total protein, albumin, globulin, alanine aminotransferase, alkaline phosphatase (ALKP), total bilirubin, cholesterol (CHOL), amylase (AMYL), and lipase levels, were measured using a VetTest chemistry analyzer (IDEXX; Westbrook, ME, USA).

### 4.4. Histological Analysis

Tissue samples (5 × 0.5 cm) of the kidney, liver, jejunum, gastrocnemius muscle, and adipose tissue were collected from each rat on day 49 of the experiment. For histological processing, tissues were fixed with 10% NBF, dehydrated, and cleared through a graded ethanol series from 70% to 100% EtOH (Sigma-Aldrich, Steinheim, Germany). The tissue samples were then embedded in xylene (Sigma-Aldrich), sectioned at 5 μm, and heated for 3 h at 45 °C on a slide warmer (77 slide warmer; Thermo Fisher Scientific, Waltham, MA, USA). The sections were deparaffinized in xylene, rehydrated in a series of graded ethanol solutions (10–70%), and rinsed with distilled water. Fibrosis and apoptosis were assessed in these tissue sections using Masson’s trichrome staining reagent and an in situ cell death detection kit, respectively. The stained specimens were examined under a microscope (Micrometrics; Nikon ECLIPSE E200, Tokyo, Japan) at 200× magnification.

### 4.5. Cecal and Fecal Content Microbial Sequencing and Data Analysis

Bacterial DNA was isolated from cecal and fecal samples using the bead-beating plus column procedure with a QIAamp DNA kit (Qiagen, Hilden, Germany) according to the manufacturer’s instructions [86]. The qualitative and quantitative analyses of the extracted DNA were conducted using 1% agarose gel electrophoresis and a microplate reader, respectively (Infinite M NANO, Tecan, South Korea). Amplification of the 16S rRNA genes was conducted in the V3-V4 regions. The specific sequences of these primers are as follows: forward primer 341F (5′-CCTACGGGNGGCWGCAG-3′) and reverse primer 805R (5′-GACTACHVGGGTATCTAATCC-3′), as previously described [87]. Libraries were sequenced on the MiSeq platform (Illumina, San Diego, CA, USA). The resulting 16S rRNA sequences were cleaned and analyzed using QIIME 2 and MicroBiomeAnalyst (2.0). Sequences were obtained from Macrogen (Daejeon, South Korea). A QIIME 2 analysis was conducted using a 2021.8 version of the software developed by Bolyen et al. [88]. A plugin (DADA2) was used for adapter and chimera removal, quality filtering, denoising, and merging [89]. An analysis was used to assess the microbial diversity and taxonomy. The amplicons were taxonomically classified using the SILVA database (version 138). Alpha diversity indices were evaluated based on the ASV tables. Additionally, the beta diversity of the fecal microbiota among the four treatment groups was analyzed using principal coordinate analysis (PCoA) of Bray–Curtis matrices.

### 4.6. Metabolite Preparation and Analysis of Blood, Liver, Kidney, Cecum, and Feces

Blood and tissue metabolomic analyses were performed as previously described [25]. For the metabolite analysis, 100 µL of serum was mixed with 400 µL of cold acetone, and the mixture was then stored in the refrigerator. The mixture was shaken for an hour using Rotamix-SLRM1 (Seoulin Bioscience Co., Seongnam-si, South Korea). Then, 400 µL of the supernatant was extracted, vacuum-dried and reconstituted in 100 µL of 20% methanol with terfenadine (Merck Millipore, Seoul, South Korea) as the internal standard. The solution was analyzed using UPLC-Q-TOF MS (Waters, Milford, MA, USA). Liver, kidney, cecal contents, and fecal samples were dissolved in 80% methanol with terfenadine for the metabolomic analysis. The samples were injected into an Acquity UPLC BEH C18 column using a mobile phase composed of water and acetonitrile at 0.35 mL/min. Blood samples took 12 min, with a column maintained at 40 °C; all other samples took 16 min. The compounds were detected using Q-TOF MS in ESI mode, with TOF-MS data scanned over *m*/*z* 100–1500 in 0.2 s. The capillary and sample cone voltages were 3 V and 40 V. The desolvation flow rate, desolvation temperature and source temperature were 800 L/h, 300 °C, and 100 °C. Leu-enkephalin ([M + H] = 556.2771) was analyzed at 10 s intervals. A QC sample prepared from all the samples was checked at regular intervals every 10 runs. The MS/MS spectra were acquired at a 10–45 eV collision energy within 50–1500 *m*/*z*. The data were processed using MarkerLynx 4.1, which enabled calculations of the mass-to-charge ratios and ion intensities. The MarkerLynx program was used for data acquisition and alignment with the following settings: peak-to-peak noise filter, noise elimination, 5% width, and an intensity threshold of 10,000. Data were aligned using a 0.05 Da mass window and 0.2 min retention window, and then normalized to standards. ChemSpider, HMDB, METLIN, and the relevant literature were used for metabolite identification.

### 4.7. Statistical Analysis

The LC-MS data were statistically analyzed using SIMCA-P+ version 12.0.1 (Umetrics, Umeå, Sweden). Partial Least Squares Discriminant Analysis (PLS-DA) was used to visualize the results, which were evaluated using R2X, R2Y, Q2, and permutation tests. A permutation test was conducted to validate the PLSDA results. Additionally, the relative abundance of metabolites was evaluated by one-way analysis of variance (ANOVA) with Duncan’s test (*p* < 0.05) using SPSS 17.0 (SPSS Inc., Chicago, IL, USA). The differential abundance of taxa among the four treatment groups was analyzed using the linear discriminant analysis (LDA) effect size (LEfSe) (LDA score >3). Growth performance and biochemical analyses were compared among the four treatment groups by analysis of variance (ANOVA) with Tukey’s test using the statistical software Prism (version 9.5.1; GraphPad Software, San Diego, CA, USA) employed for this purpose. Linear regression analysis was used to investigate the relationships between the final body weight, biochemical parameters, metabolites, and the ratio of microbiota abundance. Beta diversity and functional genetic profiles were compared among the four treatment groups using permutational multivariate analysis of variance (ANOVA) with PAST3 and 9999 random permutations. The results are expressed as means and standard errors of the means (SEMs). The difference between the control and treatment groups was statistically significant (*p* < 0.05).

## 5. Conclusions

The present study demonstrated that high-dose (≥10 mg/L) DON exposure caused adverse effects on rats, including a reduced final body weight. This high exposure also induced significant dose-dependent histological changes, such as fibrosis and apoptosis, in the liver, kidney, jejunum, muscle, and adipose tissue. Notably, the high-dose group exhibited a decline in the abundances of Firmicutes in both the cecum and feces, whereas those of Bacteroidota and Campylobacterota increased. These contrasting trends observed in the microbiome may serve as indicators of DON-induced toxicity.

## Data Availability

Datasets are available upon request from the authors.
